# Ecological dominance, functional roles, and biosafety of *Trichoderma* spp. as a biofertilizer fungus

**DOI:** 10.3389/ffunb.2025.1641004

**Published:** 2026-01-12

**Authors:** Thein Theint Win, Bo Bo, Sikandar Khan, Pengcheng Fu

**Affiliations:** 1 Food Technology Center, Naypyitaw State Polytechnic University, Naypyitaw, Myanmar; 2 Central Research Center, Yangon Technological University (YTU), Yangon, Myanmar; 3 Shaheed Benazir Bhutto University, Sheringal, Pakistan; 4 State Key Laboratory of Marine Resource Utilization in South China Sea, Hainan University, Haikou, Hainan, China

**Keywords:** agro-ecological, bioactive, biofertilizer, ecotoxicological, *Trichoderma* spp.

## Abstract

*Trichoderma* spp. are the most widely used fungal species in biofertilizers due to their capacity to enhance soil quality, suppress plant pathogens, and promote plant growth. However, due to the popularity of *Trichoderma* spp., usages in agricultural systems have raised significant environmental and safety concerns. This review mainly emphasizes the mechanisms that underlie the ecological dominance and competitive nature of *Trichoderma* spp. over native microbial communities and then explores the multifunctional role of *Trichoderma* spp. in soil ecosystems, which mainly focus on its interactions within the rhizosphere that influence dynamics plant–microbe interactions and nutrient cycling. This article also highlights potential ecological imbalances associated with prolonged or repeated applications of *Trichoderma* spp. which include changes in soil microbial biodiversity and the decline of beneficial native microbiota. Furthermore, it evaluates the safety issues of *Trichoderma*-based biofertilizers by focusing their bioactive metabolites and potential effects on humans, animals, and non-target living things. Therefore, the review addresses the importance of site-specific application strategies, monitoring protocols, and comprehensive ecotoxicological assessments to mitigate unintended environmental and health concerns. By synthesizing recent findings and identifying key knowledge gaps, this work provides a framework for the responsible and sustainable integration of *Trichoderma* spp. into modern agroecological systems.

## 1 Introduction

As sustainable agriculture becomes more widely recognized, microbial inoculants, especially fungal biofertilizers such as *Trichoderma* spp., have gained significant attention due to their multifunctional roles in promoting plant health, suppressing soil-borne pathogens, and enhancing nutrient bioavailability. They are initially characterized by their mycoparasitic and opportunistic plant-symbiotic behavior (Weindling, 1934; Howell, 2003; Harman, 2006). Therefore, *Trichoderma* spp. have been widely studied and commercialized as biocontrol agents and plant growth promoters (Harman, 2000; Vinale et al., 2008). In agricultural biotechnology, their genetic adaptability, rapid colonization ability, and capacity to synthesize diverse bioactive compounds have recognized *Trichoderma* spp. as one of the most well-documented fungal genera (Mukherjee et al., 2022).

Despite these benefits, the ecological consequences of *Trichoderma* spp. particularly in relation to their dominance mechanisms and interactions within complex soil microbial networks still remain underexplored. Amendment based on high-population single-species inoculants like *Trichoderma* spp., unlike microorganisms that have coevolved naturally within ecosystems, may disrupt long-term ecosystem functions, biodiversity resilience, and microbial balance (Barea et al., 2005). Their enhancement in fast nutrient uptake, production of antagonistic metabolites, and aggressive colonization of the rhizosphere may negatively impact native microbes involved in vital ecological processes such as organic matter decomposition, mycorrhizal symbiosis, and nitrogen fixation (Shahriar et al., 2022; Asghar et al., 2024).

Some *Trichoderma* spp. are compatible with beneficial soil organisms, whereas others can exert antagonistic effects, potentially disturbing microbial networks and altering the stability of soil trophic systems (McLean et al., 2014; Macías-Rodríguez et al., 2020; Zhang et al., 2023). These dynamics necessitate a careful assessment of how *Trichoderma* spp.-based amendments impact soil microbial community compositions, surpass local fungi and bacteria, and impact soil enzymatic activities and biochemical cycles (Shahriar et al., 2022).

Human and animal health are also another safety issues that extend beyond soil ecology. *Trichoderma* spp. produce secondary metabolites which are generally regarded as safe including peptaibols, gliotoxins, and polyketides, which exhibit bioactivity and, in some cases, cytotoxicity. These compounds are essential for pathogen suppression; their persistence and effects on non-target organisms require more detailed studies, especially under field application (Sivasithamparam and Ghisalberti, 2002; Yao et al., 2023; Han et al., 2024).

This review mainly explores the ecological predominance of *Trichoderma* spp., assessing on their interactions with native microbial populations while highlighting biosafety issues related to their extensive use. By integrating the molecular biology perspective, soil science, and microbial ecology, this paper presents a balanced review of the opportunities and limitations of *Trichoderma* spp. in sustainable agriculture. It also warns the need for systematic application strategies that promote synergy rather than microbial monopolization; ensuring the long-term sustainability of soil ecosystems.

## 2 Ecological dominance and microbial interactions

The use of *Trichoderma* spp. as biocontrol agents and biofertilizers has gained much attention due to their effectiveness in promoting plant growth and suppressing phytopathogens. However, recent research evidence indicates that their ecological dominance can induce changes in native soil microbial communities. These alterations may result in microbial imbalances that negatively impact soil health and long-term ecosystem stability. A recent study by Gao et al. (2023) demonstrated that seed treatment with *T. viride* significantly reduced the disease index of soybean root rot while altering the microbial community structure in the rhizosphere. Notably, *T. viride* increased the complexity and stability of microbial co-occurrence networks which define the extent and level of interactions among different microbial taxa. Elevated network complexity commonly reflects stronger ecological interactions and greater community stability. On the other hand, Trabelsi and Mhamdi (2013) highlighted that even though such shifts may benefit plant health, this kind of microbial inoculation may also disrupt existing microbial interactions. *Trichoderma* spp. and other microbial inoculants generally promote soil microbial biomass; however, a meta-analysis of 335 independent studies indicated that these treatments significantly impact microbial community structure. In particular, they tend to simplify bacterial network complexity, thereby compromising the resilience of soil ecosystems (Li et al., 2024).

Moreover, *Trichoderma* spp. synthesize a variety of antimicrobial secondary metabolites, notably gliotoxins and peptaibols, each exhibiting distinct modes of action. Gliotoxins inhibit fungal growth by inducing oxidative stress and disrupting enzymatic activities, whereas peptaibols create ion-permeable channels in cellular membranes, resulting in cell death. Although these metabolites enhance pathogen suppression, they may also negatively impact non-target beneficial organisms and leading to reduced microbial diversity and altered community functions (Shahriar et al., 2022). Repeated applications of *Trichoderma* spp. have been shown to change the structure and functionality of indigenous microbial communities which lead to potential long-term consequences. Such disruptions can limit beneficial microbiota, deteriorate the nutrient cycling system, and reduce soil fertility (Xiong et al., 2017; Lourenço et al., 2018). Meanwhile, inoculation with *T. harzianum* in sweet sorghum was found to increase the abundance of nitrogen-cycling genes, including those associated with nitrification and nitrogen fixation (Wei et al., 2023). This indicates a possible benefit in enhancing nutrient availability, while simultaneously highlighting the significant impact of *Trichoderma* spp. on soil microbial functionality. The increased expression of nitrogen-cycling genes indicates enhanced nitrogen mineralization and release into the rhizosphere, potentially leading to improved growth of crops and less demand on synthetic nitrogen fertilizers.

Field-based studies also report that *Trichoderma*-amended biofertilizers can significantly alter fungal communities, while exerting comparatively weaker effects on bacteria. This is likely because *Trichoderma* produces antifungal metabolites that target chitin-rich fungal cell walls, whereas bacteria possess peptidoglycan-dominated cell walls that are less responsive to these compounds. As these fungi are critical to nutrient cycling and plant health, such selective pressure may disrupt ecological balance (Hang et al., 2022; Wang et al., 2023). Up to this point, the long-term effects of *Trichoderma* spp. inoculation still remain unclear. Some studies report permanent alterations in microbial compositions, whereas others observed only temporary changes. Both of these study outcomes could affect key soil functions such as nitrogen fixation and organic matter decomposition (Trabelsi and Mhamdi, 2013).

Based on these findings, assessing the ecological risks of *Trichoderma* spp. applications is an emerging challenge to researchers. A balanced approach should consider their benefits in disease suppression and plant growth along with their influence on indigenous microbial populations. Alternative approaches such as utilizing native *Trichoderma* spp., monitoring microbial dynamics, and co-applying diverse microbial inoculants may help to mitigate ecological disturbances while enhancing sustainable soil health. Microbial shifts can be tracked using PCR-DGGE fingerprinting, qPCR-based quantification of key functional genes, 16S/ITS sequencing, metagenomics profiling, enzyme-linked functional assays, phospholipid fatty acid (PLFA) profiling, microbial respiration assays, etc., which provide insight into structural and functional changes in the soil community and allow for early detection of microbiome shifts.

**Figure 1:**
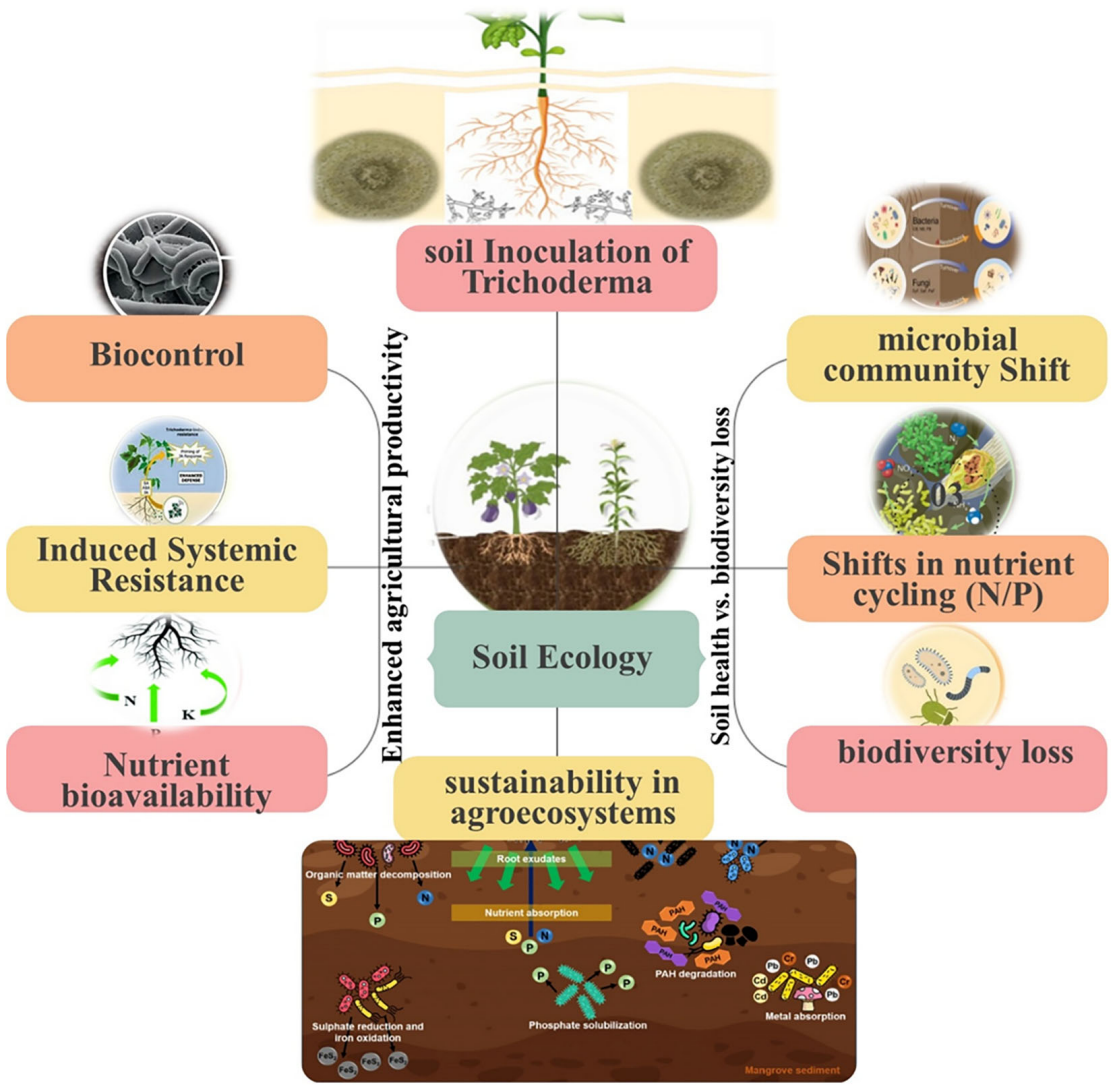
Effect of *Trichoderma* spp. inoculation on agricultural production and soil ecosystem.

**Table 1** provides a comprehensive overview of key *Trichoderma* spp. used as biofertilizers and biocontrol agents, summarizing their major functional traits, ecological roles, and reported applications in agricultural systems. It highlights their diverse mechanisms of action, including mycoparasitism, induction of systemic resistance, nutrient solubilization, and production of growth-promoting metabolites while also listing target pathogens and crop systems where they have demonstrated efficacy. This tabulated information serves as a useful comparative reference for understanding the spectrum of *Trichoderma* activity and their potential utility under different agroecological conditions. Complementing this, **Figure 1** illustrates the multifaceted interactions between *Trichoderma* spp., plants, pathogens, and the soil microbiome. It visually demonstrates how *Trichoderma* spp. colonizes the rhizosphere, suppresses pathogens through enzymatic degradation and secondary metabolites, enhances nutrient availability, and stimulates plant defense responses. Overall, readers can view **Figure 1** as a simplified map showing how *Trichoderma* spp. strengthens plant growth by simultaneously improving nutrient access and weakening disease pressure. Together, **Table 1** and **Figure 1** provide an integrated perspective on the functional versatility of *Trichoderma* spp., emphasizing both their practical benefits in sustainable agriculture and their broader ecological significance.

**Table 1:** Comparative ecological effects of *Trichoderma* spp. on soil microbial communities.

No.	*Trichoderma* species	Target microbial group	Observed effect	Reference
1.	*T. viride*	Rhizosphere bacteria	Decreased diversity	(Gao et al., 2023)
2.	*T. harzianum*	Nitrogen-fixing bacteria	Increased nifH gene copy number	(Wei et al., 2023)
3.	*T. guizhouense*	Fungal communities	Reduced AMF germination	(Islam et al., 2021)
4.	*T. koningii*	Soil fungi	Suppression of pathogenic fungi	(Monaco et al., 1991)
5.	*T. atroviride*	Soil fungi	Mycoparasitism and competition	(Benítez et al., 2004)
6.	*T. longibrachiatum*	Soil bacteria	changes the composition of the soil bacterial and fungal communities	(Yu et al., 2023)
7.	*T. reesei*	Cellulolytic microbes	Competition for cellulose	(Druzhinina and Kubicek, 2016)
8.	*T. asperellum*	Soil pathogens	Biocontrol activity	(Zapata-Sarmiento et al., 2020)
9.	*T. virens*	Rhizosphere microbes	Modulation of microbial community	(Fiorentino et al., 2018)
10.	*T. pseudokoningii*	Soil fungi	Antagonism against pathogens	(Monaco et al., 1991)
11.	*T. harzianum*	Various plant pathogens	Modulates secondary metabolite production based on pathogen type	(Vinale et al., 2009)
12.	*T. harzianum, T. atroviride*	Multiple phytopathogens	Produces bioactive compounds with antifungal activity and ISR stimulation	(Vinale et al., 2006)

## 3 Biocontrol mechanisms and agroecological functions

The colonization of *Trichoderma* spp. at the rhizosphere, a dynamic zone of soil influenced by plant root exudates and microbial activities, is complex. These fungi exhibit both beneficial and potentially suppressive effects on native microbial communities. This dual behavior raises critical questions about whether *Trichoderma* spp.-based amendment applications support a sustainable balance between pathogen suppression and microbial symbiosis.

Controlled experiments and meta-analyses consistently demonstrate that *Trichoderma*-based formulations enhance nutrient uptake, increasing root biomass. They further demonstrate a consistent reduction in disease incidence across a wide range of crops (Yedidia et al., 2001; Verma et al., 2007). These effects are attributed to their induction of systemic resistance, production of phytohormones such as auxins and gibberellins, and solubilization of nutrients like phosphorus (Contreras-Cornejo et al., 2009).

According to anonymous studies, *Trichoderma* spp. enhance plant health through various mechanisms that have been well studied. They can secrete a wide range of bioactive secondary metabolites, including terpenoids and non-ribosomal peptides, which can stimulate plant growth and induce systemic resistance (ISR) to phytopathogens (Contreras-Cornejo et al., 2016; Macías-Rodríguez et al., 2020). These fungi are also known for producing cell wall lytic enzymes and antifungal compounds, such as peptaibols and polyketides, which actively suppress soil borne diseases in the rhizosphere (Vinale et al., 2008).

In addition to phytopathogen suppression, *Trichoderma* spp. enhance nutrient availability by solubilizing phosphorus and producing siderophores that chelate iron, thereby enhancing plant nutrient uptake. Another significant beneficial trait of *Trichoderma* spp. is their ability to colonize root surfaces and form symbiotic-like associations. This kind of interactions may result in the secretion of growth-promoting substances, including indole-3-acetic acid (IAA), siderophores, and enzymes such as phosphatases and cellulases (Harman et al., 2004). Collectively, these mechanisms improve nutrient intake, root development, and resistance to abiotic stress.

However, the addition of *Trichoderma* spp. into the rhizosphere can also exert suppressive effects on non-target beneficial microbes. Brimner and Boland (2003) and Islam et al. (2021) published that certain *Trichoderma*-secreted metabolites have been shown to inhibit the germination of arbuscular mycorrhizal (AM) fungal spores, which are important for plant nutrient uptake, soil aggregation, and plant health. These adverse interactions suggest that whereas *Trichoderma* spp. can control harmful pathogens, they may also suppress beneficial microbial symbioses.

Outcomes of *Trichoderma* spp. application rarely remain consistent and depend on environmental factors such as soil chemistry, native microbial diversity, crop varieties, and climatic conditions. This dual nature of enhancing plant health while potentially disrupting microbial equilibrium highlights the significance of the research gap that must be addressed before commercial adoption for the application of *Trichoderma*-based biofertilizers. Thus, while *Trichoderma* spp. are promising tools for advancing sustainable agriculture, their introduction into soil ecosystems should be carefully monitored to preserve the functional balance of rhizospheric microbial communities.

## 4 Ecotoxicological aspects of *Trichoderma* spp.-based biofertilizers

While *Trichoderma* spp. offer considerable benefits as biocontrol agents and plant growth promoters, their potential to influence soil microbial networks and introduce ecotoxicity raises significant concerns regarding their long-term safety and environmental sustainability. These concerns are primarily linked to the production and environmental fate of their secondary metabolites, including gliotoxins, peptaibols, and polyketides.

Gliotoxins, produced notably by *T. virens*, are known for their strong antifungal and immunosuppressive properties. Although effective in pathogen control, gliotoxins have demonstrated cytotoxic effects on eukaryotic cells, raising safety concerns for non-target organisms. The environmental persistence of gliotoxin is influenced by soil pH, moisture, and microbial activity. For example, Jayalakshmi et al. (2021) found that gliotoxin remained stable in acidic soils with low microbial activities but degraded more rapidly in alkaline soils with robust microbial communities. These findings indicate that accumulation may occur in certain environments, raising concerns for soil biodiversity and long-term ecological stability. Therefore, risk assessment frameworks are necessary to ensure safe application of *Trichoderma*-based biofertilizers.

Peptaibols are linear, amphipathic peptides rich in α-aminoisobutyric acid (Aib), synthesized non-ribosomally by *Trichoderma* spp. These compounds integrate into cell membranes and form ion channels, leading to depolarization and cell death. Although compounds such as alamethicin and trichotoxins are highly effective against phytopathogens, their broad-spectrum mode of action may also affect non-target soil organisms, particularly arbuscular mycorrhizal fungi, plant-growth-promoting rhizobacteria (PGPR), nematodes, micro-arthropods, and even mammalian cells (Daniel and Rodrigues Filho, 2007; Das et al., 2018; Contreras-Cornejo et al., 2020). Soil stability and slow degradation may further promote bioaccumulation, leading to disruptions in microbial diversity and trophic interactions. Notably, the cytotoxic behavior of trichoguizaibols A–L from *T. guizhouense* was demonstrated through controlled laboratory assays against human cancer cell lines rather than environmental exposure studies (Han et al., 2024). While this suggests potential biotechnological and pharmaceutical applications, it also raises ecological safety concerns, emphasizing the need for regulations and risk assessment in field release. Polyketides, synthesized through polyketide synthase (PKS) pathways, represent a major group of *Trichoderma*-derived secondary metabolites with wide structural and functional diversity. Compounds such as 6-pentyl-α-pyrone (6-PP), widely recognized for its strong antifungal activity, and harzianic acid, known to modulate plant root development while inhibiting competing microbes, illustrate this dual ecological role. Although these polyketides contribute to biocontrol by suppressing phytopathogens, their broad-spectrum toxicity may also affect beneficial organisms, including nitrogen-fixing bacteria and arbuscular mycorrhizal fungi. Furthermore, the persistence and potential bioaccumulation of polyketides in soil remain poorly resolved, particularly given the structural variability that can significantly alter toxicity and ecological impact (Szekeres et al., 2005; Degenkolb et al., 2008; Brückner et al., 2009; Mikkola et al., 2012). These metabolites therefore enhance *Trichoderma* competitiveness but also highlight the need for ecotoxicological assessments and regulatory oversight to mitigate unintended effects in agricultural systems.”

Polyketides, synthesized through polyketide synthase (PKS) pathways, represent a major group of *Trichoderma*-derived secondary metabolites with wide structural and functional diversity. Compounds such as 6-pentyl-α-pyrone (6-PP), widely recognized for its strong antifungal activity, and harzianic acid, known to modulate plant root development while inhibiting competing microbes, illustrate this dual ecological role. Although these polyketides contribute to biocontrol efficacy by inhibiting a range of plant pathogens, their broad-spectrum activity can negatively impact beneficial soil organisms, including nitrogen-fixing bacteria and mycorrhizal fungi. Furthermore, the long-term persistence and bioaccumulative potential of polyketides in soil ecosystems remain poorly understood, particularly given the structural variability that can significantly alter toxicity and ecological impact (Szekeres et al., 2005; Degenkolb et al., 2008; Brückner et al., 2009; Mikkola et al., 2012). Collectively, these secondary metabolites play essential roles in the ecological competitiveness and efficacy of *Trichoderma* spp. in agricultural systems. However, their bioactivity also underscores the need for robust ecotoxicological evaluations. Regulatory frameworks must consider the persistence, mobility, and non-target effects of these compounds to ensure the safe use of *Trichoderma*-based biofertilizers.

**Table 2** provides a summary of the major secondary metabolites produced by *Trichoderma* spp., their biological activities, and associated ecological concerns. Comprehensive ecotoxicological studies and risk assessment models are necessary to define safe application thresholds, environmental persistence timelines, and potential interactions with native microbiota.

**Table 2:** Secondary metabolites produced by *Trichoderma* species and the biological effects of respective metabolites.

No.	Metabolite	*Trichoderma* species	Primary function	Ecotoxic/biosafety remark	Reference
I. Antifungal and antibacterial metabolites					
1	Gliotoxin	*T. virens*	Potent antifungal; suppresses competing soil fungi	Cytotoxic to mammalian and plant cells; requires dosage control in formulations	(Jayalakshmi et al., 2021)
2	Peptaibols	*T. longibrachiatum*	Antimicrobial peptides forming transmembrane pores	May disrupt non-target cell membranes; toxicity depends on concentration	(El-Nagar et al., 2024)
3	Alamethicin	*T. viride*	Antimicrobial peptide causing ion leakage	High membrane activity; could impact beneficial soil microbes	(Aidemark et al., 2010)
4	Trichothecene sesquiterpenes	*T. cf. brevicompactum*	Antifungal secondary metabolites with high potency	Toxic to eukaryotic cells at elevated levels; monitoring advised	(Yamazaki et al., 2020b)
II. Plant growth-promoting and signaling metabolites					
5	Hydrocarbonated compound	*T. atroviride*	Modulates root growth via ethylene and auxin signaling	Not fully assessed; possible hormonal imbalance in non-target plants	(Contreras-Cornejo et al., 2015)
6	6-Pentyl-α-pyrone (6-PP)	*T. koningii*	Antifungal volatile; induces plant defense pathways	May alter rhizosphere microbial balance; eco-safe at low doses	(Ismaiel and Ali, 2017)
7	Harzianic acid	*T. harzianum*	Metal chelation; promotes root health; detoxifies heavy metals	Low toxicity; suitable for bioremediation and sustainable formulations	(Tommaso et al., 2021)
III. Cytotoxic and structural bioactive peptides					
8	Epipolythiodiketopiperazines and trichothecene derivatives	*T. cf. brevicompactum*	Cytotoxic and antimicrobial; involved in interspecies defense	High caution; trichothecene group compounds known for cytotoxicity	(Yamazaki et al., 2020a)
9	Trilongins	*T. longibrachiatum*	Ion-channel–forming peptides; cytotoxic	Disrupt mammalian cell membranes; dosage-dependent toxicity	(Mikkola et al., 2012)
IV. Multifunctional and broad-spectrum compounds					
10	Peptaibiotics	*Trichoderma* spp.	Antimicrobial peptides; suppress methanogens; enhance bioelectricity	May suppress beneficial anaerobic communities; impact limited to high concentrations	(Ray et al., 2017)
11	Multiple secondary metabolites (e.g., peptaibols, polyketides, terpenes)	*Trichoderma* spp.	Broad-spectrum antimicrobial and plant growth stimulation	May alter soil microbiota composition at high dosage	(Keswani et al., 2014)
12	Various secondary metabolites (e.g., viridins, diketopiperazines)	*T. viride* MM21	Antibacterial, antifungal, and anticancer activities	Cytotoxic to mammalian systems; formulation-dependent safety	(Shaaban et al., 2023)

**Figure 2:**
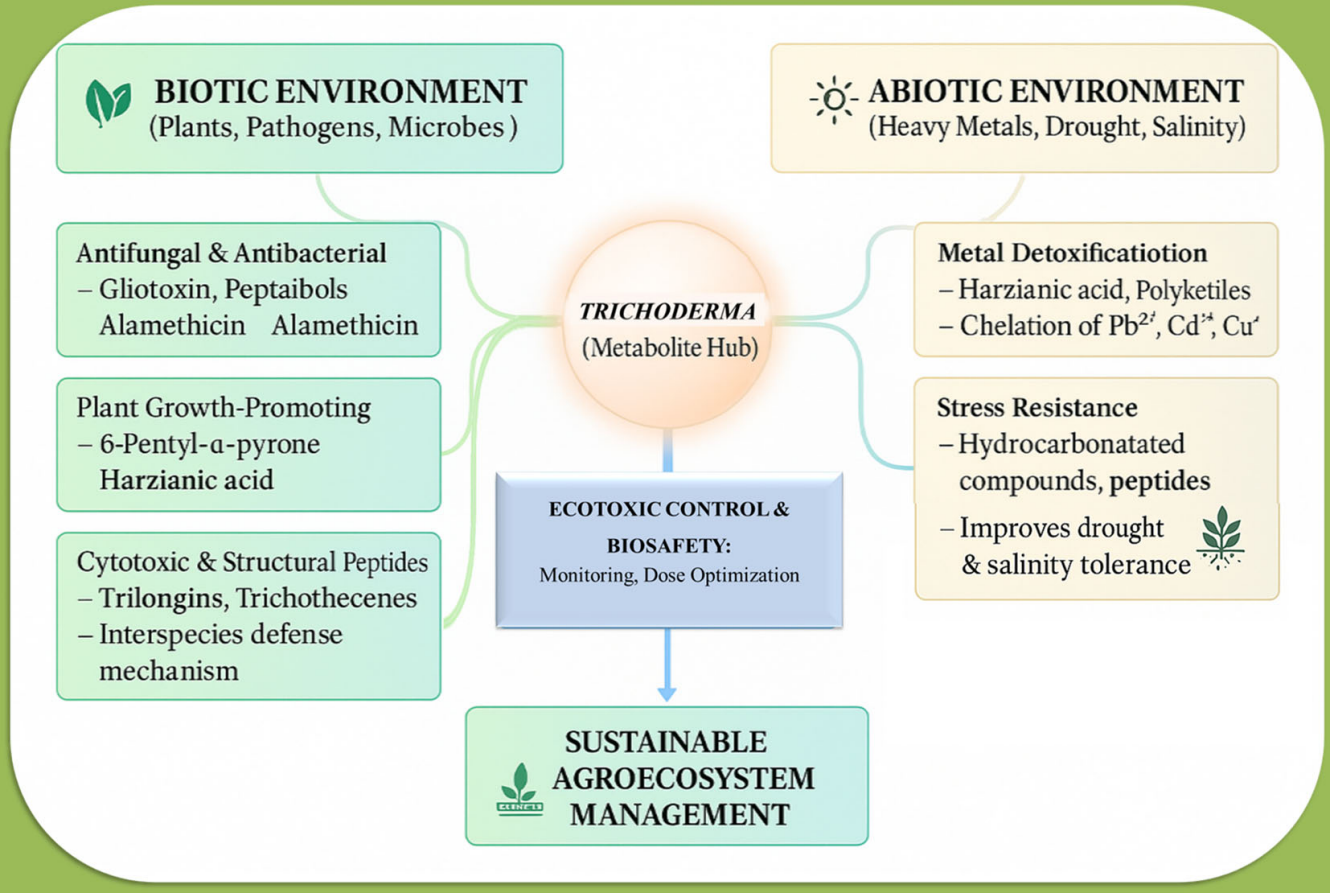
A conceptual model illustrating the interactions of Trichoderma secondary metabolites with both biotic and abiotic environments, and how these interactions support sustainable agricultural ecosystem management.

This illustration of **Figure 2** provides an extensive summary of *Trichoderma*’s role as a metabolite hub, connecting biotic and abiotic environmental components. *Trichoderma* synthesizes antifungal and antibacterial agents, including gliotoxin, peptaibols, and alamethicin, which suppress infections and preserve microbial ecology. Additional metabolites such as 6-pentyl-α-pyrone and harzianic acid promote plant growth by improving root development and systemic resistance. Cytotoxic and structural peptides, such as trilongins and trichothecenes, help regulate interspecies defense mechanisms, promoting resilience within the soil microbiome. Abiotic metabolites, like harzianic acid and polyketides, promote heavy metal detoxification by chelating detrimental ions such as Pb^2+^, Cd^2+^, and Cu^2+^. Hydrocarbon compounds and peptides enhance plant stress resistance to drought and salinity. The ecotoxicological control and biosafety framework highlights its importance of monitoring and optimizing inoculant concentrations to prevent ecological disruption or toxicity. These activities collectively enhance sustainable agroecosystem management, improving soil health, crop yield, and environmental safety.

## 5 Challenges, gaps, and future directions of trichoderma-based biofertilizers

*Trichoderma* spp. are increasingly recognized for their dual roles in biocontrol and biofertilization. Their mechanisms of rhizosphere colonization, mycoparasitism, and secondary metabolite production contribute significantly to pathogen suppression and crop yield enhancement. Their use reduces dependence on chemical fertilizers and pesticides, enhances nutrient availability, and induces systemic resistance in plants, leading to improved yields and resilience under biotic and abiotic stress (Harman et al., 2004; Lorito et al., 2010). As their field applications become widespread globally, several critical challenges and research gaps must be addressed to ensure long-term sustainability and ecological safety regarding their ecological safety, the potential for resistance development, and potential challenges to microbial community dynamics. This highlights the importance of a comprehensive study of their long-term sustainability in agroecosystems.

One of the primary limitations of *Trichoderma*-based biofertilizers is their inconsistent efficacy under field conditions. Factors such as soil type, pH, moisture, temperature, and the composition of native microbial communities significantly influence colonization success and biological activity (Hoyos-Carvajal et al., 2009). Long-term sustainability and ecological safety, critical challenges, and research gaps must be addressed through field-based validation and by developing site-specific formulations and adaptive inoculation strategies for diverse agroecological conditions. However, emerging studies highlight potential ecological risks associated with high-dose applications or the introduction of non-native *Trichoderma* spp. Soil microcosm and field trials have reported reductions in native microbial diversity, particularly among fungi and bacteria that occupy similar ecological niches (Zin and Badaluddin, 2020). Factors such as competitive exclusion, rhizosphere acidification, and the secretion of broad-spectrum antifungal compounds appear to underlie these shifts, warranting further investigation into the resilience and functional redundancy of microbial communities following repeated inoculations. Extended use of *Trichoderma* spp. against the same pathogens can lead to reduced pathogen susceptibility, a phenomenon similar to pesticide resistance. Integrating *Trichoderma* spp. into broader integrated pest management (IPM) frameworks, rotating *Trichoderma* spp. across seasons, applying inoculants in mixed formulations, or co-inoculating with beneficial bacteria (PGPR) or mycorrhizal fungi, interval-based application scheduling, and formulating consortia that distribute ecological pressure across diverse taxa, rather than relying on one dominant strain to maintain rhizosphere diversity and soil stability, are some of the methods proposed.

Resistance development is another concern. While *Trichoderma* spp. primarily target pathogens with limited adaptability, instances of reduced pathogen susceptibility after prolonged exposure have been documented (Woo et al., 2014). This phenomenon parallels challenges observed with chemical pesticides and calls for integrated pest management strategies. Current formulation technologies face several challenges, including maintaining spore viability, ensuring acceptable shelf-life, and protecting bioactive compounds during storage and transport. Approaches such as encapsulation, improved carrier materials, and co-formulation with complementary microbes may greatly enhance stability and field performance. Future research must prioritize multi-omics approaches (genomics, metabolomics, transcriptomics, and proteomics) to unravel the molecular mechanisms such as PKS/NRPS gene clusters, chitinase and glucanase pathways, siderophore biosynthesis genes, and N-cycling regulators (e.g., nifH and amoA) that underlie *Trichoderma*’s ecological interactions and functional roles. Additionally, long-term, multisite field trials are essential to assessing cumulative ecological impacts and developing best-practice guidelines for sustainable use. Regulatory frameworks should also emphasize the use of locally adapted strains, optimized dosage, and site-specific application protocols to minimize environmental risks.

## 6 Conclusion

In conclusion, while *Trichoderma*-based products offer promising tools for sustainable agriculture, offering positive impacts in biological control, nutrient cycling, and plant growth improvement, their widespread application prescribes a measured approach that balances agronomic advantages with ecological integrity. This review highlights the importance of a comprehensive evaluation of potential effects, such as unintentional alteration of native soil microbial communities and the ecological effects of bioactive metabolites. To guarantee safe and efficient application, long-term, multisite studies are essential to evaluating cumulative effects and interactions with native soil biota. The adoption of regulatory policies promoting the use of locally adapted strains, along with best practices for application timing and dosage, can help mitigate adverse impacts. Future research should incorporate ecological modeling alongside meta-omics platforms to decode how *Trichoderma* spp. regulate quorum-sensing circuits, MAP-kinase signaling cascades, oxidative stress-response genes, and root–microbe communication pathways during field establishment. Comprehensive field validation across climate zones will be essential to verifying these molecular functions under real soil conditions. With continued monitoring, *Trichoderma*-based inoculants may support the transition to climate-resilient, ecologically stable agroecosystems while minimizing unintended microbial community shift.
